# A chromo-fluorogenic HMT sensor for Ag^+^ and the resultant HMT-Ag complex turn-off probe for F^−^ in DMSO: experimental and theoretical studies

**DOI:** 10.1016/j.heliyon.2021.e06956

**Published:** 2021-05-05

**Authors:** Veikko Uahengo, Paulina Endjala, Johannes Naimhwaka

**Affiliations:** Department of Chemistry and Biochemistry, University of Namibia, 340 Mandume Ndemufayo Avenue, Windhoek, 9000, Namibia

**Keywords:** Hexamethylenetetramine, Silver and fluoride ion probe, Colorimetric sensor, Fluorometric sensor

## Abstract

The photophysical properties of Hexamethylenetetramine (**HMT**) were investigated through physical methods and spectroscopically in dimethyl sulfoxide (DMSO) at ambient temperature. Evidently, **HMT** turned out as a sensor, selective and sensitive to silver ion (Ag^+^) only, among other cations, through colorimetric and fluorometric activities (observable by naked eye) and spectrally, both by UV-Vis and fluorescence spectroscopy. The resulting complex pedant (**HMT-Ag**) is highly responsive to the presence of fluoride ion (F^−^) in aqueous soluble DMSO, evidenced by changes in absorption spectra as well as fluorescence quenching, upon addition of the respective ions. Consequently, spectral changes induced by the addition of these ions, were consistently concomitant with colour changes, from colourless to light brown (**HMT-Ag**) to dark brown (**HMT-Ag-F**) in daylight condition, while bright light blue colour (HMT) to dark blue brownish (HMT-Ag) under UV-light conditions. The experimental results were complimented by theoretical studies, which are well within agreement of one another.

## 1 Introduction

Hexamethylenetetramine (**HMT**), also known as urotropine or hexamine discovered about a century ago, is a well-known and stable chemical species, commonly used for urinary tract infection therapy. Over the years, its uses has been expanded to a wide variety of applications such as in the production of curing agents, nitrilo-triacetic acid, explosives, biocides and foodstuff industry; the practice which had since been discontinued due to its association with carcinogenicity and chronic toxicity [1, 2, 3, 4, 5, 6]. In other dimensions, due to its structural mark to adopt copious coordination binding modes (μ1 to μ4 modes), **HMT** has been employed in structural designs of coordination polymers of different dimensionalities [7]. Furthermore, **HTM** has been used as a capping agent and a surfactant in the synthesis of zinc oxides of varying nanostructure dimensions [8, 9].

Molecular recognition is a union of two or more species through non-covalent interaction modes such as metal coordination, π-staking, electrostatic force and so on, which are key building blocks of guest-host interactions. Thus, the recognition of guest chemical species by a host molecular entity, induce changes in both chemical and physical properties of the discriminating system, normally quantified through signal transducers [10, 11, 12, 13]. Since **HMT** is characterized by a hollow-cavity with N-donor atoms which is an active platform for molecular recognition, it is only logical to explore the possible interaction properties with cationic and anionic species, normally through colorimetric activities, observable by naked eyes. Colorimetric detection of cations and anions has been gaining popularity in recent times, ascribed to its simplicity and convenience in application [14, 15]. Notwithstanding, the interaction of **HMT** with cation species has been barely reported in literature, however, its coordination complexes have been synthesized, studied and reported [16, 17, 18, 19, 20].

Alternatively, fluoride (F-) ion is very vital in human physiological system, occasionally applied in the dental care fields for osteoporosis treatment. However, once the concentration of F^−^ is over the threshold, it becomes highly toxic to the physiological environment, eventually leading to osteosclerosis, fluorosis and other deadly neurodegenerative diseases [21, 22]. Thus, easy-to-synthesize and cost-effective colorimetric probes for discriminating F^−^, still remains high in the priority list. In addition, literature has recorded few reports on dual or ditopic probes sensitive and selective only to Ag^+^ and F^−^. Moreover, recognition of anions by tributary complexes of sensors has seen few reports in literature thus far, with only a few numbers available. In most occurrences, secondary recognition of anions by secondary (tributary) complexes is normally encountered in reversible sensing systems, with a good record in literature so far, however with few to none for Ag^+^/F- modulated system. Thus, secondary recognition of anions through secondary complexes presents a new dimension towards molecular recognition mechanisms [23, 24, 25, 26, 27, 28, 29, 30, 31, 32, 33, 34].

**Figure 1:**
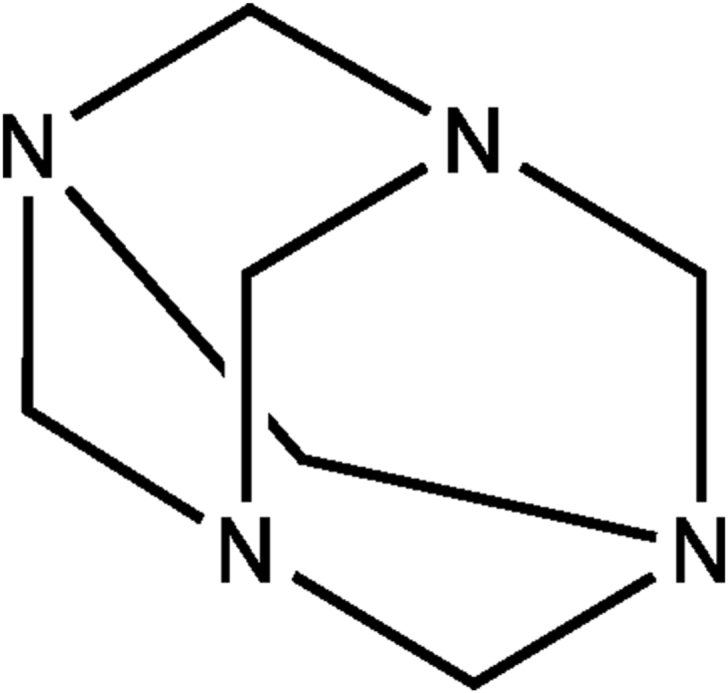
The molecular structure of **HMT**.

Moreover, reports have indicated the synthesis of stable silver (I)- **HMT** (Htm-Ag)-based coordination compounds, which have been crystalized and studied [16, 17]. However, only a few transition metal complexes with **HMT**, as a ligand, have been reported or studied to-date, hence the motivation for this particular study. Herein, the study focuses on the molecular recognition of **HMT** (Figure 1) and its pedants towards anions and cations, via the process of chemosensing, in aqueous soluble dimethyl sulfoxide environments. Accordingly, no reports are available in literature, about the molecular recognition of **HMT** or **HMT-Ag** with anionic or cationic species or any application of **HMT** as a functional material.

## 2 Experimental

### 2.1 Reagents and instruments

All chemicals and reagents, including **HTM** were purchased from Sigma–Aldrich unless stated. All solvents were used as received, unless stated. Metal salts of K^+^, Li^+^, Ag^+^, Na^+^, Fe^2+^, Pb^2+^, Ca^2+^, Mg^2+^, Mn^2+^, Co^2+^, Cd^2+^, Zn^2+^, Ni^2+^, Cu^2+^, Hg^2+^, Fe^3+^, Al^3+^and Cr^3+^ (mostly chlorides and nitrate salts) were purchased from Sigma–Aldrich. While the anions of CN^−^, Br^−^, AcO^−^, H_2_PO_4_^-^, NO_3_^-^, I^−^, F^−^, OH^−^, Cl^−^, ClO_4_^-^ and HSO_4_^-^ were used as tetrabutylammonium (TBA) salts, purchased from the same supplier. All UV-Vis analysis were measured on the Perkin Elmer Lambda 35 spectrophotometer in a 3.0 ml quartz cuvette with 1cm path length. The fluorescence analysis was performed on a Molecular Device spectarMax M2, primarily for Plate Reader usage. The density functional theory (DFT) calculations at B3LYP/6-31G∗∗ were performed using a Spartan ’14 package software [35].

### 2.2 Methods used for UV-Vis and Fluorescence analysis

All UV-Vis analysis and titration were performed on a Perkin Elmer Lambda 35 spectrometer using DMSO as a solvent, by adding standard TBA solutions (0.03 M), while the concentration of **HMT** (1 × 10^−5^ M) was kept constant all along. The anion solutions (CN^−^, Br^−^, AcO^−^, H_2_PO_4_^-^, NO_3_^-^, I^−^, F^−^, OH^−^, Cl^−^, ClO_4_^-^ and HSO_4_^-^) and cations K^+^, Li^+^, Ag^+^, Na^+^, Fe^2+^, Pb^2+^, Ca^2+^, Mg^2+^, Mn^2+^, Co^2+^, Cd^2+^, Zn^2+^, Ni^2+^, Cu^2+^, Hg^2+^, Fe^3+^, Al^3+^and Cr^3+^ (as chlorides and/or nitrate salts) were used for analysis in the UV-Vis and Fluorescence experiments. The emission spectra were performed in the same way as the absorption spectra on a Molecular Device spectarMax M2 instrument, with the same concentrations of host (guest) and guest (anions).

## 3 Results and discussions

### 3.1 Colorimetric investigation of **HTM** with cations

**Figure 2:**
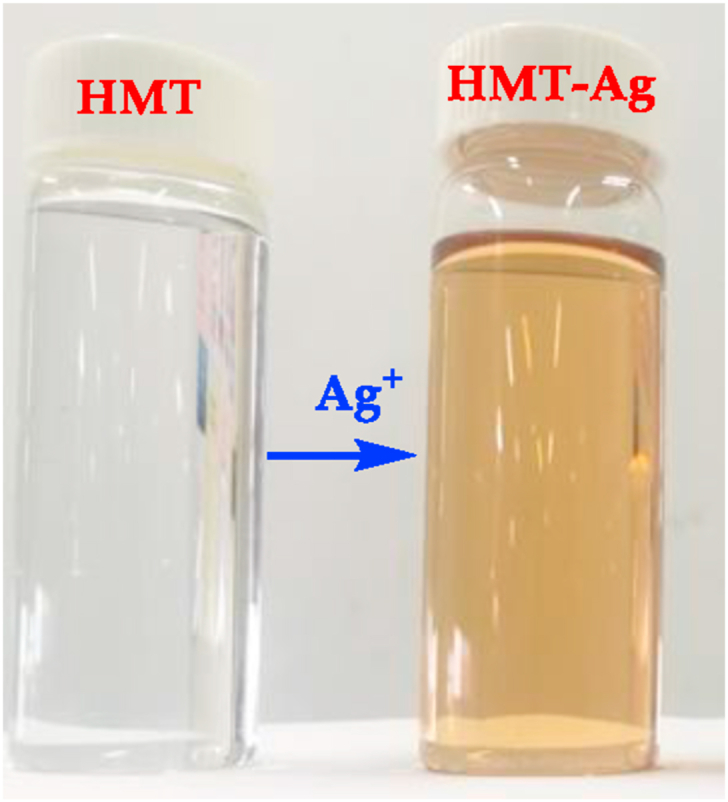
Colour activities of **HMT** (1 × 10^−3^ M) at ambient temperature, upon the addition of Ag^+^, thus **HMT-Ag**, both in DMSO.

Comprehensive studies of the interactions between the host (**HMT)** and guest species (cations), the prepared metal salt solutions (0.03 M, in DMSO) were titrated against **HMT** (1 × 10^−3^ M), each separately. The results were recorded as observed (Figure 2) accordingly. The sequential molar addition of Ag^+^ (AgNO_3_) to **HMT**, resulted in observable visual changes (Figure 2), signaling the formation a **HMT-Ag** pedant, appearing steady and gradual, in a delayed effect behavior-like. The intensity of the colours observed was concentration-dependent, displaying intense colour (at higher concentration) to lighter colours (at lower concentrations). The colorimetric activities are ascribed to the coordination induced union between **HMT** and **Ag**^**+**^, resulting in a **HMT-Ag** pedant according to literature [16, 17]. Interestingly, the molar additions of other standard metal salt solutions used, did not induce any significant visual changes, upon adding them to **HMT**.

### 3.2 Colorimetric investigation of **HMT-Ag** complex with anions

**Figure 3:**
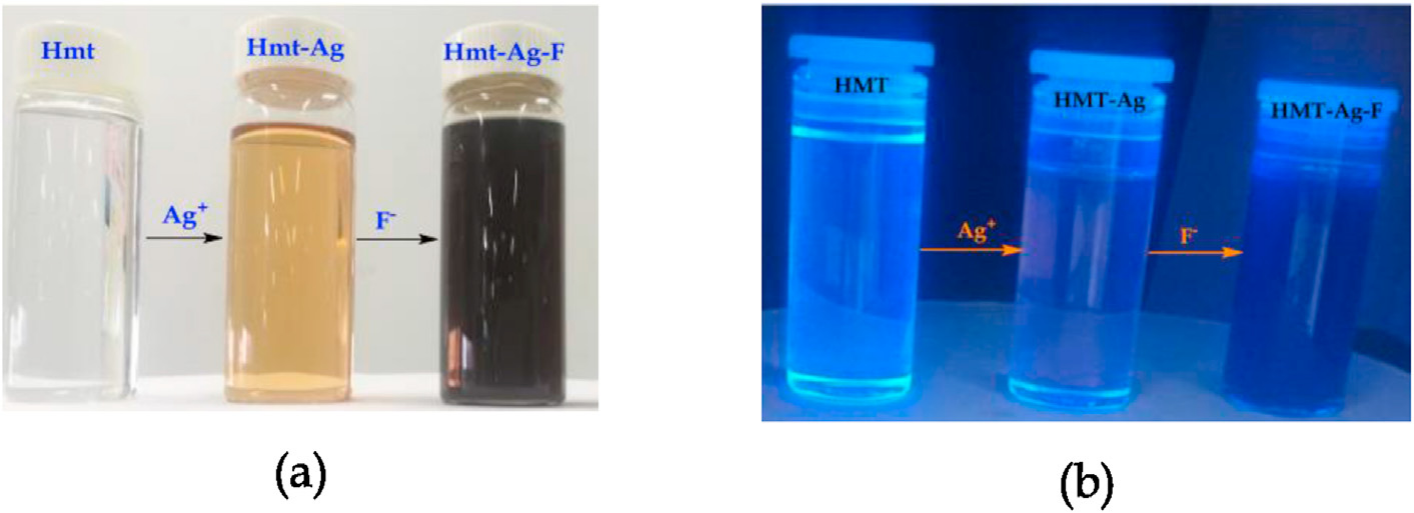
Visual colour changes upon titration of F^−^ with **HMT-Ag** in DMSO (1 × 10^−3^ M) under, (a) daylight and (b) UV-light conditions, at room temperature.

Accidently, while discarding the waste solutions of **HMT-Ag** and those of anions in a collective waste container, we noticed and observed some colorimetric activities taking place within the waste container. Thus, this triggered the investigation to study the chemical relationship between the **HMT-Ag** complex and each anion. Interestingly, upon the addition of F^−^ (as a TBAF) to **HMT-Ag**, a more intense, visible colour change was observed from the light brown to dark brown (Figure 3). The change in colour is due to the chemical unison between **HMT-Ag**. In addition, under UV-light conditions, HMT displayed similar patterns to daylight conditions, except that the colorimetric activities observed were from light blue (**HMT**), blue-brownish (**HMT-Ag**) and dark blue (**HMT-Ag-F**), as displayed (Figure 3a and b). The colour changes are normally indicative of the existence of chemical interactions among the participating species, which are complimented spectroscopically. Contrastingly, apart from F^−^, other anions have not displayed any noticeable colour changes upon the addition to the solution of **HMT-Ag** complex.

### 3.3 The photophysical properties of **HMT**

**Figure 4:**
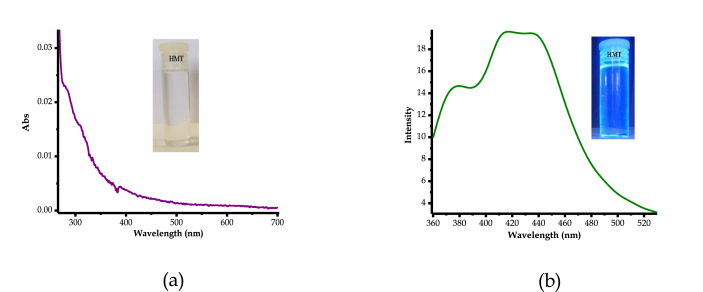
Optical behaviors of **HMT** (1 × 10^−5^ M), (a) Absorption spectra, (b) Emission spectra (*λ*_ext_ = 300 nm).

The characteristics of the UV-Vis spectra of **HMT** in DMSO were defined by an intense band, extended from 250 to 400 nm (Figure 4a), corresponding to π→π∗ electronic transitions due to internal charge transfer (ICT) of the **HMT**. Moreover, **HMT** displayed a prominent fluorescent emission characterized by three low energy vibronic bands at 380 nm, 415 nm and 434 nm with the maximum emission displayed at 415 nm and 434 nm (Figure 4b), when excited at 300 nm. In addition, under UV-light and daylight conditions, **HMT** displayed distinctive colours, ranging from colourless (Figure 4a inset) to light blue (Figure 4b inset), respectively. The light blue colour under UV-vis is suggestive that **HMT** has fluorescent emission properties, just as observed spectroscopically.

### 3.4 Photophysical behaviours of **HMT** upon interacting with cations and anions

**Figure 5:**
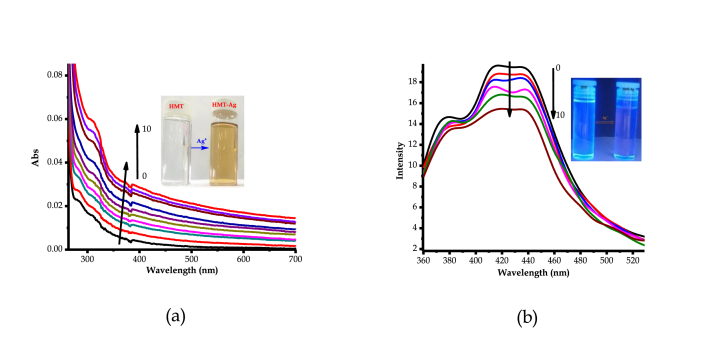
Spectral behaviours of **HTM** (1 × 10^−5^ M) with Ag^+^ in DMSO, (a) UV-Vis spectra, (b) Emission spectra (300 nm_exc._), both at room temperature.

Comprehensively, in order to put colorimetric behaviors of **HTM** with cations into perspective as observed (Figure 3), detailed UV-Vis and fluorescence experiments were performed. The titration of Ag^+^ (AgNO_3_) with **HMT**, in DMSO, resulted in spectral shift activities, as displayed (Figure 5a). The gradual addition of Ag^+^ resulted in the enhancement (hyperchromic shift) of absorption spectra of **HMT** between 300 nm and 700 nm, which is accompanied by the change in colour (colourless to light brown), ascribed to the coordination induced charge transfer (Figure 5a inset). The coordination induced charge transfer resulted in the formation of a **HMT-Ag** complex formed, thus light brown colour formed. The formation of the complex which is concomitant with colorimetric activities, which is in conformity with the earlier proposed polymeric structure reported in literature [16, 17]. Predictively, the communication is of coordination nature, through the nitrogen donor atoms from of **HMT**, attributed to the complementarity properties of the host-guest unison. After the molar addition of up to 10 equiv. of Ag^+^, no spectral or colorimetric changes could be further observed, as the system seemed to have reached saturation. The addition of other cations or anions carried out could not induce any significant changes, both colorimetrically and spectrally.

Similarly, fluorescence studies of **HMT** with Ag^+^ were conducted in the DMSO solution. As displayed (Figure 5b), **HMT** showed a fluorescence emission characterized by three low energy vibronic bands at 380 nm, 415 nm and 434 nm with maxima emission positioned between 415 nm and 434 nm. Subsequently, the molar addition of Ag^+^ to **HMT** (1 × 10^−5^ M) in DMSO had a quenching effect on the fluorescence emission, ascribed to the formation of the **HMT-Ag** pedant via coordination induced interaction. The fluorescence emission properties are supported by the intense light-blue colour of **HMT**, which upon interacting with Ag^+^, experienced a reduced intensity as well as a slight colour change (to dark bluish) as displayed (Figure 5b inset). Moreover, after the molar addition of 10 equiv. of Ag^+^ to **HMT**, no significant quenching changes were spectrally detected and observed any longer, even when huge amounts were added, signifying the saturation or equilibrium effect on **HMT**.

### 3.5 Theoretical studies of **HMT** and **HMT-Ag**

**Figure 6:**
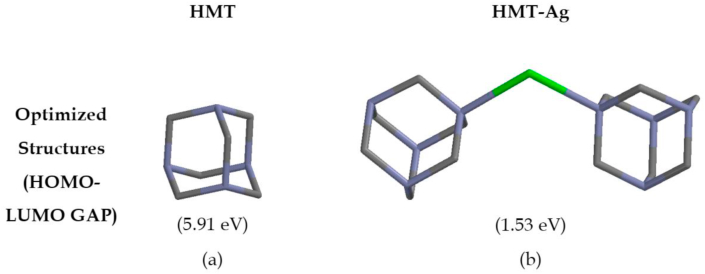
Optimized structures of (a) **HMT** and (b) Proposed **HMT-Ag**, with estimated HOMO-LUMO energy gaps.

**Figure 7:**
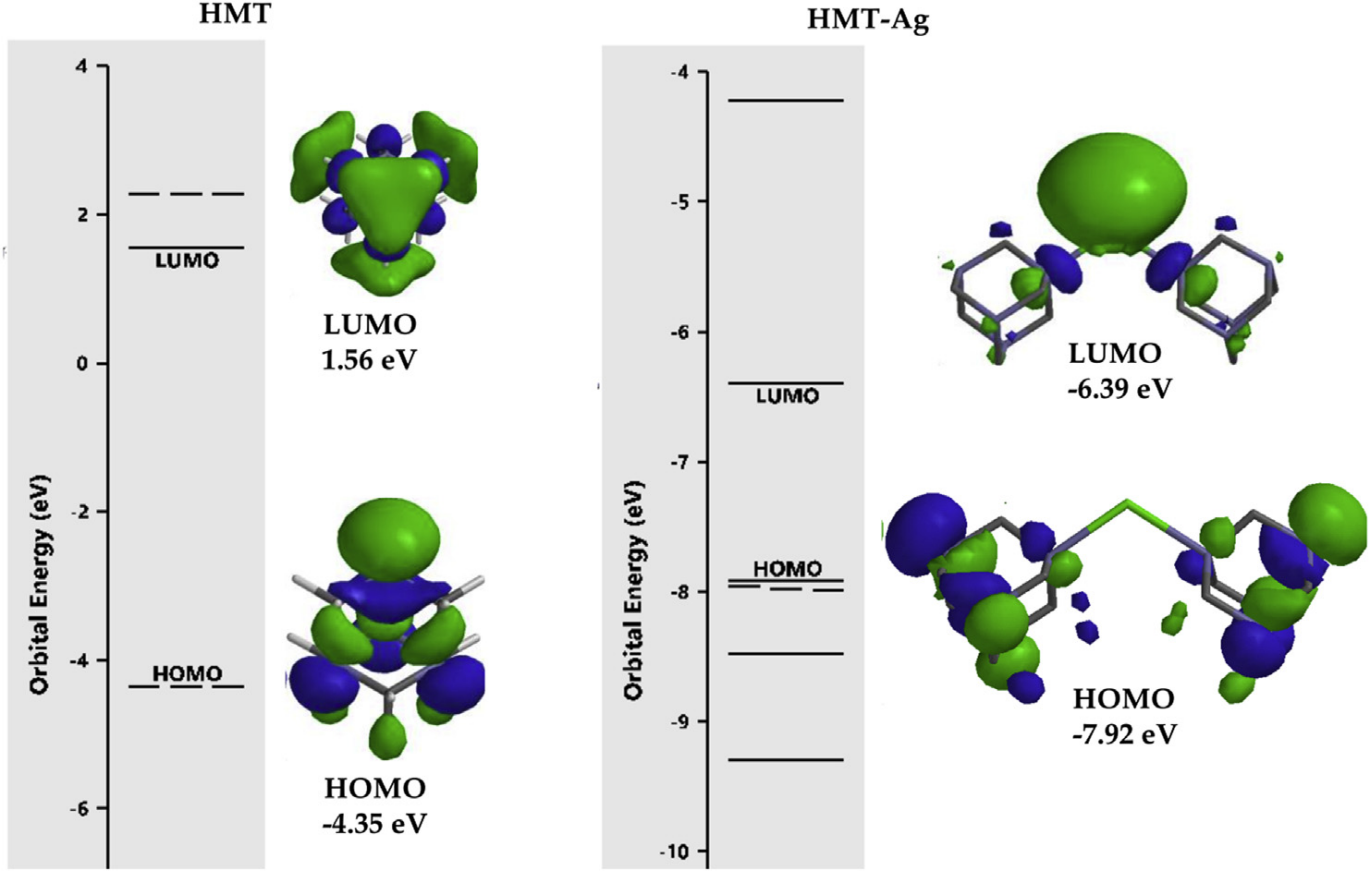
HOMO and LUMO diagrams of **HMT** and **HMT-Ag**.

To complement the experimental data obtained, theoretical studies using DFT program at B3LYP/6-31G∗∗ (Spartan ’14 package) level in the default solvent were performed, to predict the optimized structural orientation of **HMT** and its resultant complex **HMT-Ag** (Figure 7) [36]. Theoretically, the computed energy gap of **HMT** was found to be 5.91 eV, the information extracted from the LUMO and HOMO values [37, 38, 39, 40]. However, upon introducing the Ag^+^ to **HMT**, the system experienced a significant decrease in the HOMO-LUMO gap by predictably forming **HMT-Ag**, with the new energy gap of 1.53 eV (Table 1). Evidently, the energy gap of **HMT** before and after binding was 5.91 eV and 1.53 eV respectively, significantly decreasing upon binding with Ag^+^ (Figure 6a and b). The proposed and predicted interaction between **HMT** and Ag^+^ is shown (Figure 6b), as suggested in literature, via crystal structure studies performed previously [21, 22].

**Table 1:** HOMO-LUMO data of **HMT** and interaction with Ag^+^.

	HMT (eV)	HMT-Ag (eV)
LUMO	1.56	−6.39
HOMO	−4.35	−7.92
**Energy Gap**	**5.91**	**1.53**

Contrastingly, the change in spectral data, from the absorption and fluorescence, these actions have been complemented by theoretical data, where the decrease in HOMO-LUMO gap is symbolic towards the formation of a more stable complex (**HMT-Ag**). Furthermore, the variation of molecular orbitals in **HMT** and its complex, are testament to the predicted interaction nature of the two species. In **HMT**, the concentration of the HOMO lies along the electron donor sources of nitrogen (N), while the LUMO lies more along the three carbon atoms of the chair-like conformation. In the complexed state (**HMT-Ag**), the concentration of the HOMO lies within the **HMT** ring, while the LUMO are predominantly localized on the silver atom (Figure 7).

### 3.6 Solvatochromic studies on the optical and electronic properties of **HMT**

**Figure 8:**
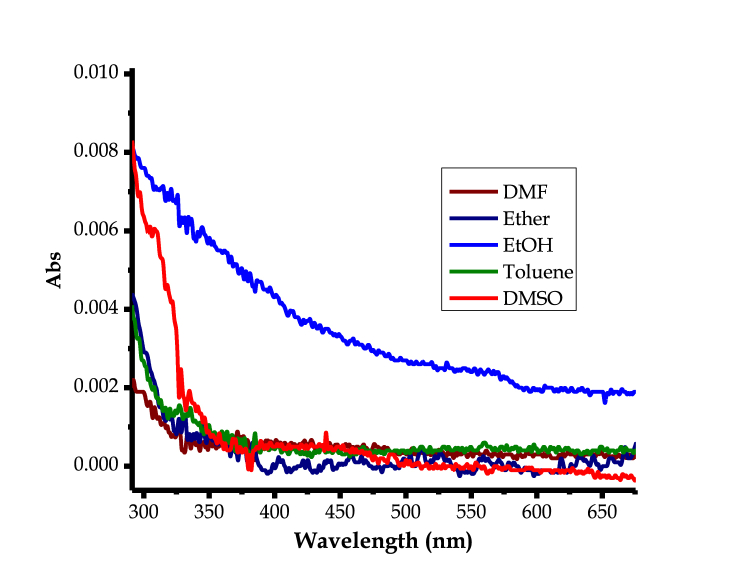
Solvatochromic effects on absorption spectra of **HMT** (1 × 10^−5^ M) in different solvents (inset).

The optoelectronic properties of **HTM** in solvents of varying polarities were investigated, studied and compared, both experimentally and theoretically. Optoelectronic properties are generally determinants of energy gaps, which can easily predict appropriate applications of any molecular entity. Herein, it is evident that **HMT** behaves differently in solvents of varying polarities, as displayed by the absorption spectra data (Figure 8). In Table 2, **HMT** has displayed varying absorption patterns, even though all activities are within the UV-region. It is clear from the spectra, that **HMT** absorbs slightly different in ethanol comparing to all other solvents used (Figure 8), as shown by the enhanced molar absorptivity coefficient. The optical properties in different solvents are more or less similar, as indicated by the spectra and the respective electronic properties (Table 2).

**Table 2:** Theoretical electronic property data of **HMT** in different solvents.

Solvent	E_HOMO_	E_LUMO_	E_GAP_
Ether	−4.34 eV	1.56 eV	5.90 eV
CH_2_Cl_2_	−4.36 eV	1.55 eV	5.91 eV
Toluene	−4.33 eV	1.55 eV	5.88 eV
EtOH	−4.38 eV	1.52 eV	5.90 eV
THF	−4.35 eV	1.56 eV	5.91 eV
DMF	−4.35 eV	1.56 eV	5.91 eV

**Figure 9:**
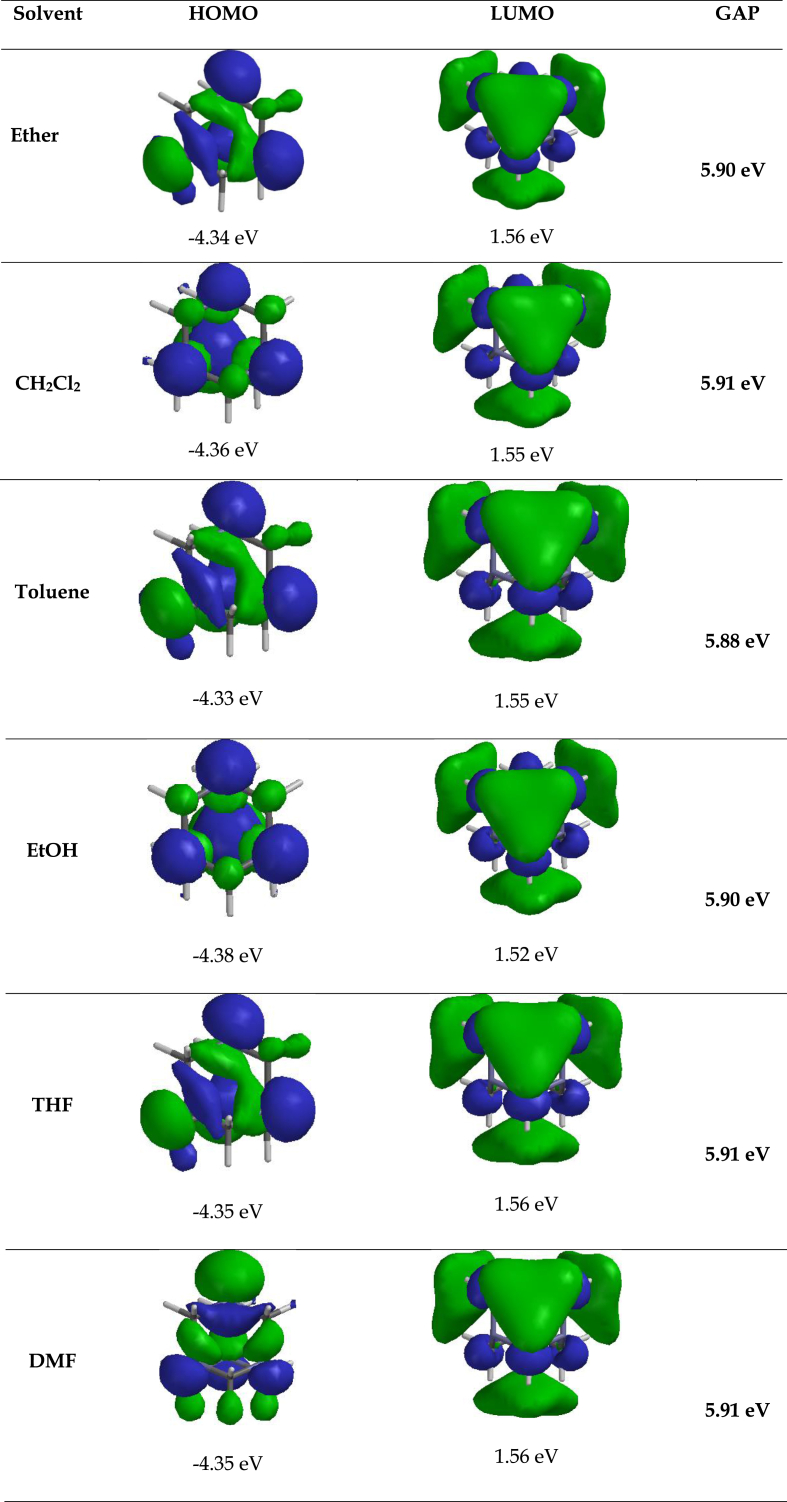
Frontier molecular orbitals of **HMT** modelled in different solvents.

Furthermore, Table 2 displays energy gap information in different solvents as estimated from the theoretical data. Most of the computed energy band gaps of **HMT**, are within 5.90 eV in different solvents, are well in agreement with the experimental UV-vis spectral data, which are all predicting that the molecule absorbs mostly in the ultraviolet region of the spectrum (≥3.1–124 eV). The variance in absorption spectra of **HMT** in different solvents is minimal, with only toluene displaying a slightly different HOMO-LUMO gap (5.88 eV). In addition, the frontier orbitals distribution were also investigated, in different solvents (Figure 9), whereby the HOMO are concentrated on the nitrogen donors in the molecular framework, while the LUMO are clouding on the three axial carbon planes, the trend observed in all solvents.

In addition, the association of **HMT-Ag** is presumably via coordination through the nitrogen atoms in a proposed 1:2 (**Ag-HMT**) interaction mode as indicated (Figure 6b). In some instances, the interaction could take place via the hollow cavity of **HMT**. However, in this case, the hollow cavity of **HMT** is too small in volume, with a diameter of 2.834 Å, to engulf the atomic size of Ag^+^ (with the ionic radius of 1.7 Å), more or less of its own size. Moreover, the chemical environment of the cavity mouth of **HMT** does play a vital role into the interaction between the two species. Guided by supramolecular phenomenon of self-assembly through weaker Van Der Waals force, **HMT** is only harmonious with Ag^+^ among all transition metal cations used, by displaying colorimetric activities once Ag^+^ is introduced. This has indeed confirmed the early studies, which succeeded in crystalizing **HMT-Ag** out of solutions, among all metals used. In rare studies, it has been reported that some group metals do form complexes bearing HMT as a ligand [41, 42, 43, 44, 45], however, no colorimetric activities were observed.

### 3.7 UV-Vis titrations of **HMT-Ag** with F^-^

**Figure 10:**
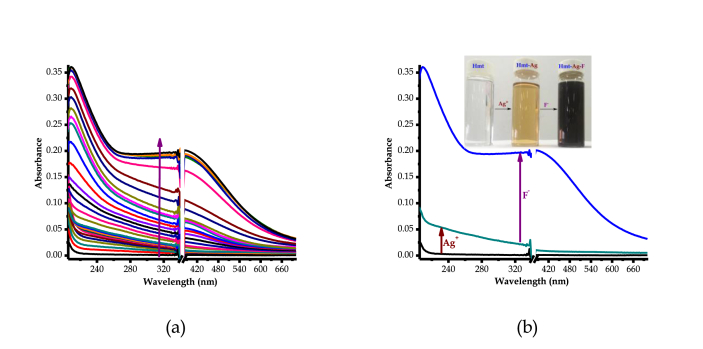
UV-Vis spectra of **HMT-Ag-F** (1 × 10^−5^ M) in DMSO, (a) inclusive spectra up to 10 equiv. Ag^+^ and 30 equiv. addition of F^−^, (b) only spectra of Ag^+^ (10 equiv.) and F^−^ (30 equiv.), at room temperature.

Broadly, to further understand the interaction of the **HMT-Ag** pedant and the anions, as confirmed by colour changes above, spectroscopic analysis were conducted through the titrations of the complexed pedant (**HMT-Ag**) with F^−^ in DMSO solution. Firstly, the molar titration of up to 10 equiv. of Ag^+^ to **HTM**, resulted in hyperchromic shift accordingly, till no noticeable spectral changes were observed anymore. However, the introduction of the molar amount of F^−^ to **HMT-Ag** caused even more hyperchromic shift, simultaneously shadowed by subsequent colorimetric activities, signaling chemical interactions on the two entities (Figure 10a). After the addition of up to 20 equiv. of F^−^, a broad absorption band with peaking at 486 nm extended into the visible region started to gradually appear. The absorption band grew more intense as more F^−^ quantities of up to 30 equiv. were added. The inter-spectra correlation between **HMT-Ag** and **HMT-Ag-F** with respect to added molar quantities of Ag^+^ and F^−^ are streamlined (Figure 10b). In short, it appeared that, the addition of up to 10 equiv. of Ag^+^ to **HMT**, the system reached the saturation point, that no observable changes could be detected anymore, however, on further molar addition of F^−^ to a saturated solution of **HMT-Ag**, spectral enhancement were observed again accompanied by colorimetric activities (Figure 10b inset). The interaction of F^−^ with **HMT-Ag** has triggered the fluorescence emission turn-on effect which resulted in the further enhancement of fluorescence, the same trend observed in the absorption spectra (Figure 10).

### 3.8 Fluorescence studies of **HMT-Ag** with F^-^

**Figure 11:**
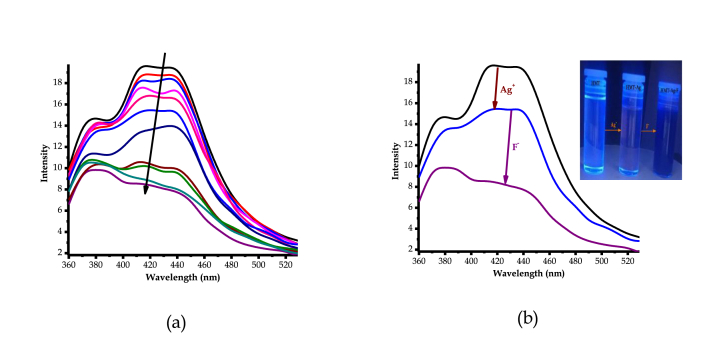
Fluorescence spectra of **HMT-Ag-F** (1 × 10^−5^ M) in DMSO, (a) inclusive spectra up to 10 equiv. Ag^+^ and 30 equiv. addition of F^−^, (b) only spectra of Ag^+^ (10 equiv.) and F^−^ (30 equiv.) each, at room temperature.

Factually, absorption properties are closely associated to fluorescence behaviours in a particular molecular entity; the emission properties of **HMT-Ag-F** were studied at the excitation wavelength of 300 nm, in DMSO, as in UV-vis studies (Figure 11a). The molar additions of F^−^ to **HMT-Ag** resulted in even more emission quenching of the complex, as compared surpassing the effect of Ag^+^ (Figure 11b). The fluorescence quenching of **HMT-Ag** upon adding F^−^, is also reflected by the colorimetric behaviours under UV-light conditions, where the characteristics of a bright blue colour of **HMT** has diminished significantly to dark bluish colour (Figure 11b inset). The change in colorimetric activities (diminishing brightness) is demonstration to added fluorescence quenching, as the case of the complex. Obviously, the fact that no interactions was observed, both colorimetrically and spectrally, upon the addition of F^−^ to **HMT** (in the absence of Ag^+^), is evident that the coordination of Ag^+^ to **HMT** plays an intermediate role of inducing pedant functional recognition of fluoride ions. Thus, **HMT-Ag** complex can be classified as a colorometric and fluorogenic probe for fluoride ions in DMSO.

### 3.9 Detection of F^−^ in real samples using **HMT-Ag**

**Figure 12:**
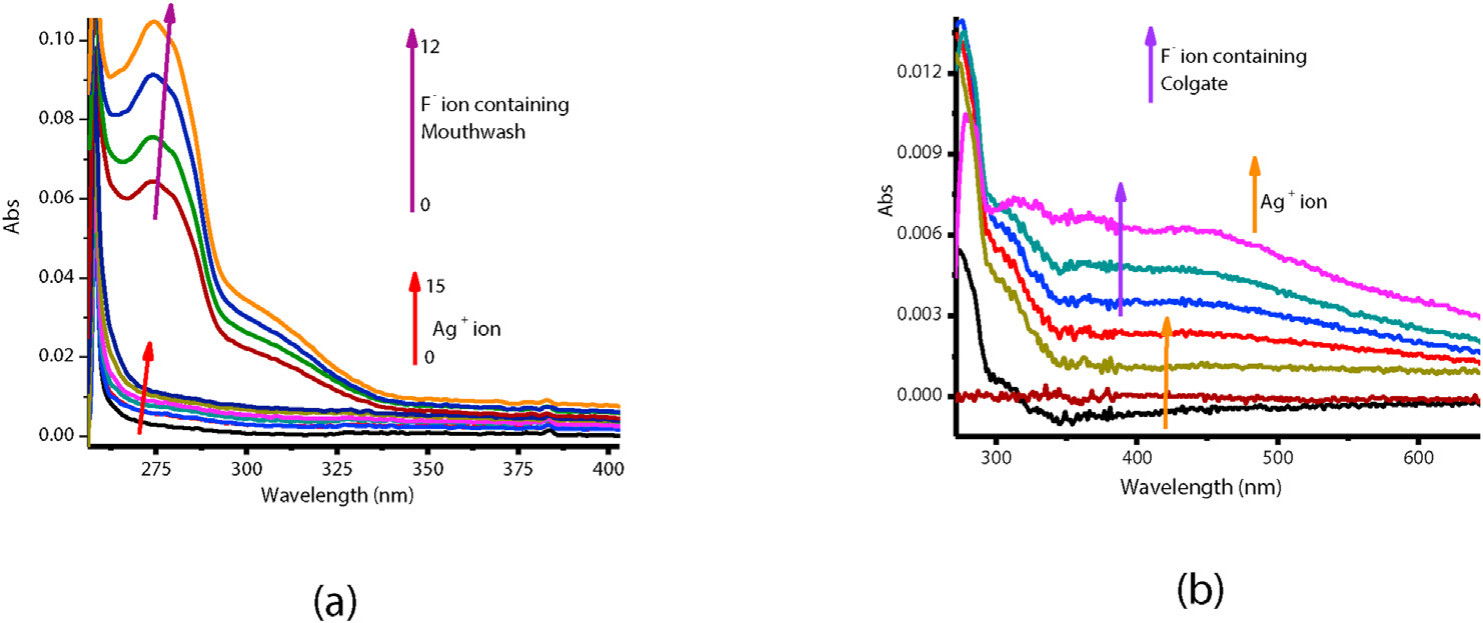
UV-Vis titration spectra of **HMT-Ag** (1 × 10^−5^ M) in DMSO of (a) Mouthwash (12 equiv.), (b) Colgate (15 μL), toothpastes dissolved in H_2_O each.

The experiment on real time application was investigated and carried out, to test for the efficacy of **HMT-Ag** as a fluoride sensor, by applying quantitative analysis using commercially obtained Colgate and a Mouthwash, all known to contain fluoride ions. The preparation of a toothpaste sample solution was prepared according to a known method, as 20 mg/ml in 1 ml of H_2_O. The Colgate aqueous solution was titrated against **HMT-Ag** (1 × 10^−5^ M in DMSO) in molar quantities as shown (Figure 12). The sequential molar addition of a mouthwash solution to **HMT-Ag**, resulted in the spectral changes, clearly displaying that there exists a chemical interaction between **HMT-Ag** and F^−^ ions in the mouthwash (Figure 12a). The spectral patterns and behaviours of a mouthwash and Colgate are quite similar to **HMT-Ag** vs F^−^ (Figure 10a). The well-defined spectral response of **HMT-Ag** vs mouthwash, demonstrates the high impact of fluoride ions in the mouthwash, as compared with Colgate (Figure 12b). Consequently, the definition and resolution of the spectra demonstrated that the concentration of fluoride ion in mouthwash is higher than that of Colgate, especially considering that similar quantities were used. Thus, **HMT-Ag** complex can quantitatively discriminate fluoride ions in environmental samples of aqueous nature. Thus, **HMT-Ag** complex displayed convincing evidence that it can be a worthy addition to the body of knowledge, especially considering that **HMT** is a highly stable compound with very little known applications in material science.

## 4 Conclusion

Conclusively, the chemosensing properties of **HMT**, a commercially available chemical, have been investigated and studied, towards cations and anions in aqueous soluble DMSO. The discrimination of a biologically important Ag^+^ among other cations, both colorimetrically and spectrally, was investigated and confirmed. More importantly, the **HTM-Ag** pedant turned out to selectively and sensitively discriminate F^−^ among other anions, observable through colorimetric activities as well as fluorescence quenching. The discrimination of F^−^ is uniquely impended in the secondary interaction, a rare occurrence in the field of chemical sensing. The discrimination of F^−^ was also observed in real sample applications of a Colgate and mouthwash studied, which proved to be a deserving addition to the body of existing knowledge in this field. Thus, **HMT** and **HMT-Ag** are very significant additions to the body of chemosensing for both Ag^+^ and F^−^ in literature, as well as to more understanding the properties and functions of a rarely researched Hexamethylenetetramine.

## Declarations

### Author contribution statement

Veikko Uahengo: Conceived and designed the experiments; Analyzed and interpreted the data; Wrote the paper.

Paulina Endjala: Performed the experiments.

Johannes Naimhwaka: Analyzed and interpreted the data.

### Funding statement

This work was supported by the Research and Publication Unit (No: URPC/2014/153) of the University of Namibia, Namibia.

### Data availability statement

Data included in article/supplementary material/referenced in article.

### Declaration of interests statement

The authors declare no conflict of interest.

### Additional information

No additional information is available for this paper.
